# Role of Intracellular Lipid Logistics in the Preferential Usage of Very Long Chain-Ceramides in Glucosylceramide

**DOI:** 10.3390/ijms17101761

**Published:** 2016-10-21

**Authors:** Toshiyuki Yamaji, Aya Horie, Yuriko Tachida, Chisato Sakuma, Yusuke Suzuki, Yasunori Kushi, Kentaro Hanada

**Affiliations:** 1 Department of Biochemistry and Cell Biology, National Institute of Infectious Diseases, 1-23-1 Toyama, Shinjuku-ku, Tokyo 162-8640, Japan; horietty0315@gmail.com (A.H.); tachida@nih.go.jp (Y.T.); sakumac@nih.go.jp (C.S.); hanak@nih.go.jp (K.H.); 2 Department of Materials and Applied Chemistry, College of Science and Technology, Nihon University, Chiyoda-ku, Tokyo 101-8308, Japan; suzuki.yuusuke@nihon-u.ac.jp (Y.S.); kushi.yasunori@nihon-u.ac.jp (Y.K.)

**Keywords:** genome editing, sphingolipid, ceramide synthase, glucosylceramide, sphingomyelin, CERT

## Abstract

Ceramide is a common precursor of sphingomyelin (SM) and glycosphingolipids (GSLs) in mammalian cells. Ceramide synthase 2 (CERS2), one of the six ceramide synthase isoforms, is responsible for the synthesis of very long chain fatty acid (C20–26 fatty acids) (VLC)-containing ceramides (VLC-Cer). It is known that the proportion of VLC species in GSLs is higher than that in SM. To address the mechanism of the VLC-preference of GSLs, we used genome editing to establish three HeLa cell mutants that expressed different amounts of CERS2 and compared the acyl chain lengths of SM and GSLs by metabolic labeling experiments. VLC-sphingolipid expression was increased along with that of CERS2, and the proportion of VLC species in glucosylceramide (GlcCer) was higher than that in SM for all expression levels of CERS2. This higher proportion was still maintained even when the proportion of C16-Cer to the total ceramides was increased by disrupting the ceramide transport protein (CERT)-dependent C16-Cer delivery pathway for SM synthesis. On the other hand, merging the Golgi apparatus and the endoplasmic reticulum (ER) by Brefeldin A decreased the proportion of VLC species in GlcCer probably due to higher accessibility of UDP-glucose ceramide glucosyltransferase (UGCG) to C16-rich ceramides. These results suggest the existence of a yet-to-be-identified mechanism rendering VLC-Cer more accessible than C16-Cer to UGCG, which is independent of CERT.

## 1. Introduction

Sphingolipids are a class of lipids containing a sphingoid base (e.g., sphingosine and dihydrosphingosine) and are essential components of cell membranes, which function in various biological events including cell growth, apoptosis, differentiation, and adhesion [1,2,3,4,5,6,7]. The *N*-acylated form of sphingosine is ceramide, the key intermediate for the biosynthesis of sphingomyelin (SM) and glycosphingolipids (GSLs) (Figure S1). The de novo biosynthesis of ceramide mainly occurs in the endoplasmic reticulum (ER), and the synthesized ceramide is then transported to the Golgi apparatus where SM and glucosylceramide (GlcCer) are synthesized. SM synthase 1, which is predominantly responsible for the de novo synthesis of SM, is localized to the medial/trans regions in the Golgi apparatus, whereas UDP-glucose ceramide glucosyltransferase (UGCG), the GlcCer synthase, is distributed to the cis/medial or earlier regions of the Golgi apparatus [8]. The ER-to-Golgi trafficking of ceramide includes at least two pathways: ceramide transport protein (CERT)-dependent and -independent ones [9,10,11,12]. CERT mediates the ER-to-Golgi non-vesicular trafficking of ceramide, which is the major pathway for the synthesis of SM but not GlcCer [9]. The CERT-dependent pathway has been well studied, whereas CERT-independent pathways including the one for GlcCer synthesis and the minor pathway for SM synthesis have not been well characterized.

In mammals, six isoforms of ceramide synthases (CERS1–6) play roles in the *N*-acylation of dihydrosphingosine to produce dihydroceramide in the de novo pathway and the *N*-acylation of sphingosine to produce ceramide in the salvage pathway [13,14]. Each CERS has a different acyl-CoA preference in the synthesis of (dihydro)ceramide. For example, CERS2 synthesizes C20–26 VLC-Cer, whereas CERS5 synthesizes C16-containing ceramides (C16-Cer) [15,16,17,18,19]. The balance of CERSs has an impact on the fatty acid composition of sphingolipids [20]. It is still unclear whether the imbalance of sphingolipid species leads to pathophysiological processes or whether it is rather the end point of a pathophysiological process. CERS2 null mouse liver contains only trace amounts of VLC-sphingolipids, but instead shows increased amounts of C16-sphingolipids, probably because other CERSs (CERS5 and 6) serve as the main CERSs in compensation [21].

Recent studies have shown that not only differences in the polar head moieties of sphingolipids but also their acyl chain length affects various cellular phenomena and signaling [22]. The binding of microorganisms to lactosylceramide (LacCer) can activate Lyn, an intracellular Src family kinase, only when the acyl chain of LacCer is a VLC (mainly C24:0 and C24:1) but not a C16 fatty acid (FA) [23]. CERS2-deficient mice, which scarcely produce VLC-sphingolipids, undergo insulin resistance and several organ defects [24,25]. On the other hand, C16:0-Cer can form channels on mitochondria to promote apoptosis, and VLC-Cer inhibits the mitochondrial permeabilization [26]. The acyl chain length of sphingolipids also affects their intracellular transport. Endocytic trafficking routes of SM species differ depending on their acyl chain length, and SM species with a longer fatty acid tend to be routed to the late endocytic pathway before their recycling to the plasma membrane [27].

It has long been known that the FA compositions of GSLs and SM are different [28,29,30], although both GlcCer and SM are synthesized from the same precursor. The proportion of VLC species in GSLs is higher than that in SM in various cell types including mouse macrophage-like RAW264.7 cells, human cervical cancer HeLa cells, and Chinese hamster ovary (CHO) cells [29,30]. Although the mechanisms underlying the different FA compositions have not been clarified, this phenomenon means that the balance of CERS expression is not the only factor that affects fatty acyl lengths in sphingolipids.

In this study, we created three types of CERS2-deficient HeLa cell mutants using a genome editing technology. These mutants included not only a knockout cell line but also mutants that expressed small amounts of CERS2 at the different levels, thus providing cognate cell lines having different proportions of VLC-sphingolipids. Using these new tools, we attempted to address the question of how the difference in FA composition between GlcCer and SM is created in living cells.

## 2. Results

### 2.1. Preparation of CERS2 Mutants in HeLa Cells by TALEN

**Figure 1:**
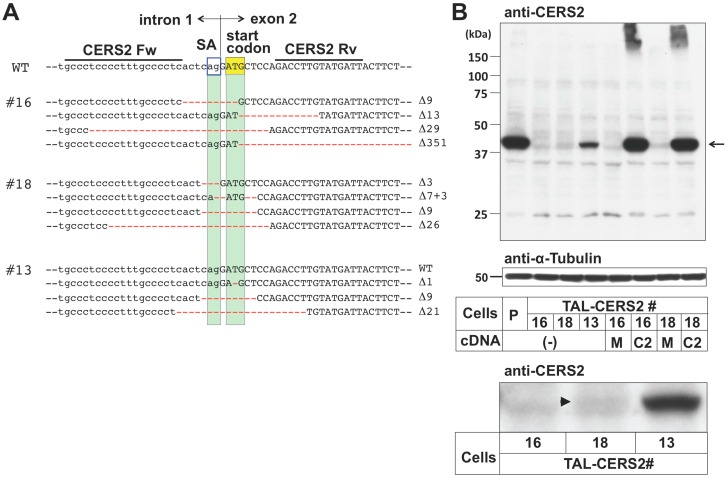
Isolation of *CERS2*-deficient clones. (**A**) The target site of the TALEN-CERS2 pair in the human *CERS2* gene and indel analysis in TALEN-CERS2–treated (TAL-CERS2) clones. The target sequences (CERS2 Fw and Rv) are located in intron 1 and exon 2, respectively. The sequence between the TALEN target sequences contains the start codon (yellow box) and the splicing acceptor (SA) at the end of intron 1 (blue lined box). Green boxes highlight the portions corresponding to the start codon and the SA. Deletions are shown in red and their lengths are specified on the right of the sequences; (**B**) Western blot analysis of CERS2 in the TAL-CERS2 clones. Cell lysates from the parent cells and CERS2-deficient mutants were immunoblotted with anti-CERS2 antibody (**top**) and anti-α-tubulin antibody (**middle**). Part of the enlarged view of the top image is shown at the **bottom**. The arrow and arrowhead indicate CERS2; P, parent cell line; 16, TAL-CERS2#16 clone; 18, TAL-CERS2#18 clone; 13, TAL-CERS2#13 clone; (-), no transfection; M, mock; and C2, CERS2 cDNA restoration. Note that overexpression of CERS2 resulted in formation of high molecular weight aggregates.

**Figure 2:**
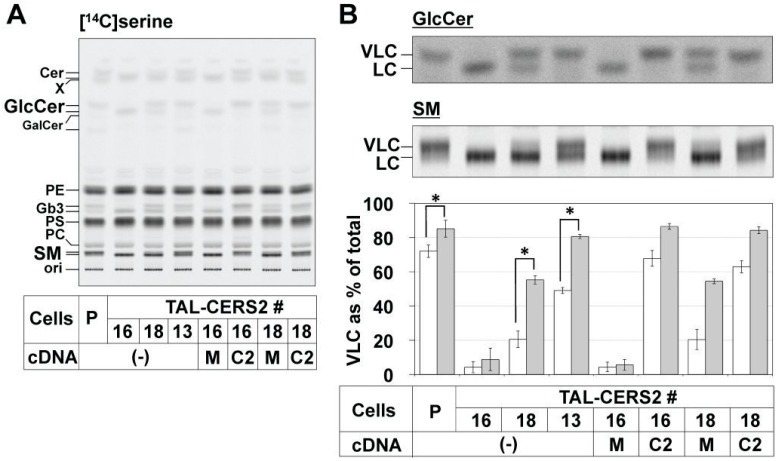
Metabolic labeling of lipids in TAL-CERS2 clones. (**A**) Metabolic labeling of sphingolipids with radioactive serine in the parent cells and TAL-CERS2 clones shown in Figure 1B. Cells were cultured with [^14^C]serine for 16 h, and lipids extracted from the cells were separated by normal-phase thin-layer chromatography (TLC) (Method 1). Radioactive image of an analyzed TLC plate is shown. Cer, ceramide; GalCer, galactosylceramide; PE, phosphatidylethanolamine; PS, phosphatidylserine; PC, phosphatidylcholine. P, parent cell line; 16, TAL-CERS2#16 clone; 18, TAL-CERS2#18 clone; 13, TAL-CERS2#13 clone; (-), no transfection; M, mock; C2, CERS2 cDNA restoration. The darker image is shown in Figure S3A; (**B**) comparison of the proportion of the upper band intensities (indicating the proportion of very long chain (VLC)) of GlcCer and sphingomyelin (SM). The enlarged and darker images of [^14^C]serine-labeled GlcCer and SM shown in (**A**) are aligned. The proportions of VLC-containing lipids are expressed as the upper (VLC-containing) band intensity as a percentage of the sum of the upper (VLC) and lower (C16 long chain fatty acid (FA) (LC)-containing) band intensities of GlcCer and SM for each cell line shown in Figure 1B: mean percentage ± standard deviation (SD) obtained from five independent experiments. The Student’s *t*-test was used. * *p* < 0.05.

We first remodeled the proportion of VLC-type sphingolipids by genome-editing of the *CERS2* gene in a HeLa cell line (HeLa mCAT#8) using transcription activator-like effector nucleases (TALEN) technology [31,32]. A TALEN target site was selected around the start codon, where the splice-acceptor (SA) on the 3’ side of intron 1 was also located (Figure 1A). The mutations of SAs often cause missplicing; therefore, a variety of transcripts were expected to be produced, which may have resulted in the creation of various types of mutants. Three clones, TAL-CERS2#16, #18, and #13, were isolated, in which three or all four of the *CERS2* gene alleles were mutated (Figure 1A). Then, the effect of these mutations on the expression levels of *CERS2* mRNA and protein was characterized in each mutant. Reverse transcription-polymerase chain reaction (RT-PCR) analysis showed that these clones expressed various kinds of transcripts (Figure S2B). In the TAL-CERS2#16 clone, the start codons of all gene alleles were mutated, and, furthermore, two of the four gene alleles lost the SA site. Mutations of the SA sites will usually cause exon skipping or a SA shift near the original SA site. Transcriptional analysis showed that all analyzed transcripts of *CERS2* in this clone lost the original start codon (Figure S2C). More than half of the analyzed transcripts showed exon 2 skipping due to the deletion of SA, as expected (Figure S2C(e)). The exon 2-skipped transcript was unlikely to be functional even though it may have been translated: although the ATG codon in exon 5 of this transcript could have been used as a new start codon, the potential translation product lost the 150 N-terminal amino acids, which are essential for the activity [33]. Most other transcripts of *CERS2* also seemed to be nonfunctional because a frame-shift occurred even if another possible start codon was used (Figure S2C(b–d)). In one transcript, an extra sequence containing a potential start codon was observed due to the SA shift, and the transcript might have translated functional CERS2 if the potential start codon were used (Figure S2C(a)). However, Western blot analysis showed that no specific band of CERS2 was detected under this condition (Figure 1B). Then, the de novo synthesis of sphingolipids was analyzed by metabolic labeling with [^14^C]serine in this clone. In thin-layer chromatography (TLC) of the metabolically labeled lipids, the upper band of SM, which was most likely to represent SM subspecies having a VLC, such as the C24:1 acyl chain (C24:1-SM) [30,34], had almost disappeared, and only the lower band (mainly C16:0-SM) was detected (Figure 2A,B, and Figure S3). These results suggested that little or no functional CERS2 was expressed in TAL-CERS2#16 cells. The clone #16 retained the normal metabolic labeling of phosphatidylserine or phosphatidylethanolamine, indicating no clear cytotoxic influence of the genome editing in the cell clone (Figure 2A).

In the TAL-CERS2#18 clone, the SA sites were mutated in all *CERS2* gene alleles. Three of the four alleles also lost the start codon or were frame-shifted, which was also confirmed in the transcriptional analysis (Figure S2D(c,d)). However, the wild-type open reading frame was preserved in one allele in spite of a deletion of the SA site on the 5′ side of the start codon. A *CERS2* transcript in this clone contained the wild-type open reading frame with the extended exon 2 sequence, which emerged due to the SA shift (Figure S2D(a)). In this transcript, the Kozak consensus sequence on the 5′ side of the original start codon was disrupted. Therefore, CERS2 proteins were expected to be less frequently translated from this transcript than from the wild-type one. By Western blot analysis, only a small amount of CERS2 was detected in this clone (Figure 1B). However, in contrast to the TAL-CERS2#16 clone, which displayed no de novo synthesis of VLC-containing SM (VLC-SM), the TAL-CERS2#18 clone substantially produced VLC-SM (~20% of total newly synthesized SM) (Figure 2B).

In the TAL-CERS2#13 clone, three alleles lost either the start codon or SA site, but one allele still contained the wild-type *CERS2* gene (Figure 1A). In this clone, CERS2 was detected by Western blot analysis, although the amount of the protein was lower than that in the parent cells (Figure 1B). Together with the results in the other clones, the amounts of CERS2 increased in the following order: #16 < #18 < #13 < the parent cell line, and these amounts were correlated with the proportion of SM species having VLC-Cer corresponding to the upper band in the TLC (Figure 2B). These reductions in the upper band intensities of SM in the TAL-CERS2#16 and #18 clones were restored by transfection with *CERS2* cDNA (Figure 1B and Figure 2, and Figure S3). In the parent cells, *CERS2* and *CERS5* mRNAs were clearly expressed, and mRNA expressions of *CERS1* and *CERS4* were also detected in RT-PCR analysis (Figure S4A). Quantitative PCR analysis demonstrated that the expression levels of *CERS1*, *CERS4*, and *CERS5* were almost identical among the established three mutant clones and the parent cell line (Figure S4B). *CERS3* was expressed only a little, and *CERS6*, which can also synthesize C16-Cer besides *CERS5*, was undetected in all established mutants (Figure S4A,C), suggesting that CERS5 was the predominant ceramide synthase in HeLa cells in the absence of CERS2 and compensated for a lack of VLC-Cer by producing C16-Cer. These results are consistent with the previous reports [34,35,36]. The TAL-CERS2#16 and #18 clones, which expressed no or a low level of CERS2, were prone to proliferate a little more slowly than the parent cells, although the difference was not statistically significant (Figure S4D).

### 2.2. Differential Usage of VLC-Cer between GSLs and SM

Next, the proportion of upper band intensity to the total band intensities in GSLs was compared with that in SM for each mutant. In TAL-CERS2#16 cells, not only SM but also GSLs including GlcCer lost the upper band, which is consistent with the disruption of all *CERS2* alleles (Figure 2 and Figure S3). On the other hand, in TAL-CERS2#18 cells, which expressed only small amounts of CERS2, the proportion of the upper band intensity in GlcCer was about 50%, which was much higher than that in SM (20%) (Figure 2). Metabolic labeling experiments with [^14^C]galactose also demonstrated that the proportion of the upper band intensity in GlcCer as well as Gb3 was about 50% in the TAL-CERS2#18 clone (Figure S5). Furthermore, in the TAL-CERS2#13 clone, the proportion of the upper band intensity in GSLs was almost the same as that in the parent cells, although the expression level of CERS2 was thought to be one fourth of that in the parent cells, and the proportion of the upper band intensity in SM was still lower (Figure 2, Figures S3 and S5). These results suggest that VLC-Cer was a precursor of GSLs more frequently than it was for SM. Even in parent cells, the difference between GlcCer and SM was statistically observed (Figure 2B), but the difference was small because the major subspecies of all sphingolipids were already the VLC-type. The TAL-CERS2#18 clone exhibited a clear difference in acyl composition between GlcCer and SM; therefore, we considered this clone to be a good cell model for the elucidation of mechanisms underlying the difference.

**Figure 3:**
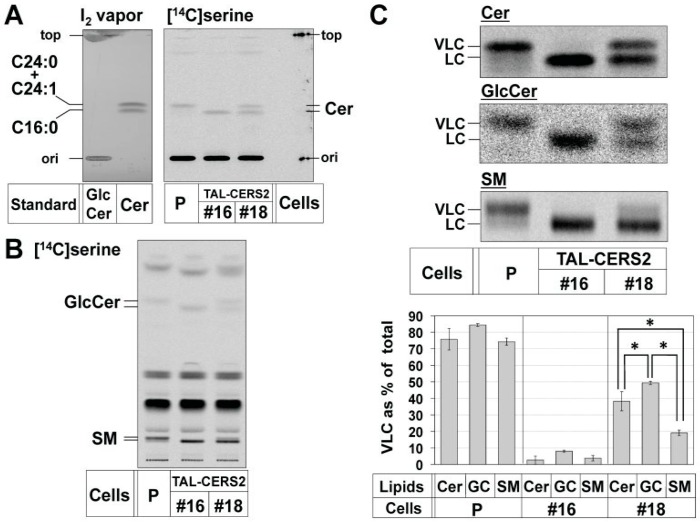
Different fatty acid preferences of GlcCer and SM. (**A**,**B**) Metabolic labeling of sphingolipids with radioactive serine for a short time. Cells were cultured with [^14^C]serine for 2 h, and lipids extracted from the cells were separated by TLC. (**A**) Separation between upper (VLC-containing) and lower (LC-containing) bands of ceramide by normal-phase TLC (Method 3 described in the “Experimental Section”). Non-radiolabeled ceramide standards (C16:0, C24:1, and C24:0) were developed simultaneously and visualized by iodine vapor. The positions of the spot origin (ori) and the solvent front (top) in two images were aligned; (**B**) separation of upper and lower bands of GlcCer and SM by normal-phase TLC according to Method 1. Radioactive images of analyzed TLC plates are shown. The darker image is shown in Figure S6B; (**C**) comparison of the proportion of upper band intensities (indicating the proportion of VLC) of ceramide, GlcCer, and SM shown in (**A**,**B**). The enlarged and darker images of [^14^C]serine-labeled ceramide, GlcCer, and SM shown in (**A**,**B**) are aligned. The proportions of VLC-containing lipids are expressed as described in Figure 2B: mean percentage ± SD obtained from three independent experiments. The Bonferroni corrected Student’s *t*-test was used for multiple comparisons. * *p* < 0.017.

To elucidate whether the differential usage of ceramides occurred during the de novo synthesis of sphingolipids, the amount of VLC species as a proportion of the total amount in each sphingolipid species (i.e., ceramide, GlcCer, and SM) was determined by metabolic labeling with [^14^C]serine within a short time (2 h) in the mutants. When using an appropriate developing solvent system in TLC, ceramides exhibited clear doublet bands (Figure 3A,C). To confirm the FA lengths in [^14^C]serine-labeled ceramides, we also analyzed the FA lengths of ceramides by the combination of normal-phase and reverse-phase TLC with standard ceramides of different acyl chain lengths (Figure S6A). The parent cells mainly synthesized C24:1- (and C22:0-) and C24:0-Cers, whereas almost all of the ceramides synthesized in HeLa TAL-CERS2#16 cells were C16:0-Cer. Thus, the upper band intensity expressed as a proportion of the total band intensities (the sum of the upper and lower band intensities) in normal-phase TLC can represent the amount of the VLC-containing lipids expressed as a proportion of the total cognates in each sphingolipid species. In the TAL-CERS2#18 clone, the proportion of VLC to the total in ceramides was about 40%, whereas the proportion in GlcCer was ~50%, and that in SM was only ~20% (Figure 3 and Figure S6B). These results indicated that the VLC-preference of GlcCer and the C16-preference of SM in their de novo synthesis were clearly observed in HeLa TAL-CERS2#18 cells. The proportion of VLC to the total in the indicated lipid was abbreviated to the “proportion of VLC-lipid”.

### 2.3. Enrichment of De Novo Synthesized C16:0-Cer in the ER Did Not Affect the Preferential Usage of VLC-Cer in GlcCer

**Figure 4:**
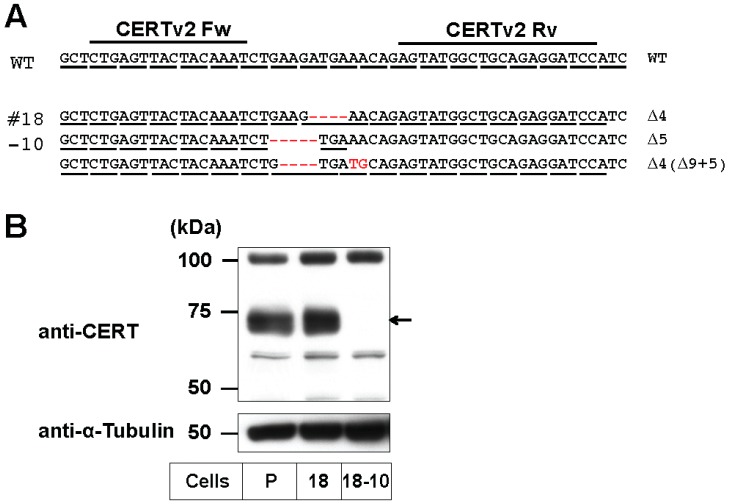
*CERT* gene disruption by the TALEN–CERT pair in the TAL-CERS2#18 clone. (**A**) The target site of the TALEN–CERT pair in the human *CERT* gene and indel analysis of the TAL-CERS2#18-CERT#10 (#18-10) clones. The target sequences (CERT Fw and Rv) are located in exon 2, which codes for part of the pleckstrin homology (PH) domain. Deletions and replacements are shown in red and their lengths are specified on the right of the sequences. WT, wild type; (**B**) Western blot analysis of CERT in the TAL-CERS2#18 clones. Cell lysates from the parent cells and the indicated mutants were immunoblotted with anti-CERT antibody (**top**) and anti-α-tubulin antibody (**bottom**). The arrow indicates CERT at 68 kD. P, parent cell line; 18, TAL-CERS2#18 clone; 18-10, TAL-CERS2#18-CERT#10 clone.

**Figure 5:**
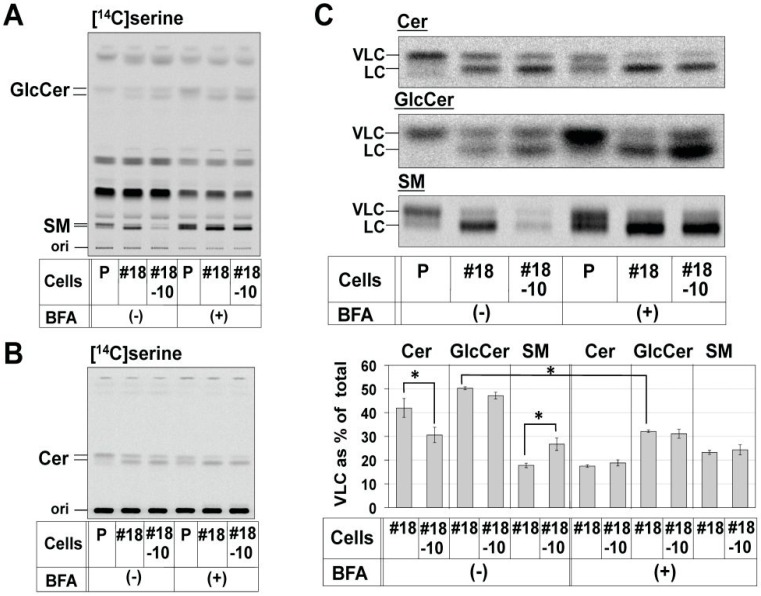
Effects of CERT disruption and Brefeldin A (BFA) treatment on the proportion of VLC species in sphingolipids. (**A**,**B**) Metabolic labeling of sphingolipids with radioactive serine in the parent cells (P), TAL-CERS2#18 clone (#18), and TAL-CERS2#18-CERT#10 clone (#18-10). Cells were cultured with [^14^C]serine in the presence (+) or absence (-) of BFA for 2 h, and lipids extracted from the cells were separated by Method 1 for GlcCer and SM in (**A**) and Method 3 for ceramide analysis in (**B**). The darker images are shown in Figures S7A and S7C; (**C**) comparison of the proportion of VLC of ceramide, GlcCer, and SM shown in (**A**,**B**). The method used is as described in Figure 2B. The enlarged and darker images of [^14^C]serine-labeled ceramide, GlcCer, and SM shown in (**A**,**B**) are aligned. The proportions of VLC-containing lipids are expressed as described in Figure 2B: mean percentage ± SD obtained from three independent experiments. The mean percentage in the TAL-CERS2#18 clone was compared with that in the TAL-CERS2#18-CERT#10 clone (#18-10) and that in the BFA-treated TAL-CERS2#18 clone, and the Bonferroni corrected Student’s *t*-test was used for the multiple comparisons. * *p* < 0.025.

The VLC-Cer–preference in GSLs was paradoxical, because ceramides are de novo synthesized in the ER as a common precursor of both GlcCer and SM. This paradox may be accounted for by the possibility that SM may preferentially use C16:0-Cer, and then, the rest of the ceramide subspecies containing a higher proportion of VLC-Cer may be non-selectively used by UGCG, thereby generating the apparent VLC-Cer–preference in GSLs. To test this, we disrupted the *CERT* gene in the TAL-CERS2#18 clone using TALEN. The gene encodes the ceramide transport protein CERT, which mediates the transport of ceramide from the ER to the Golgi site for the synthesis of SM [9,37]. An isolated clone (TAL-CERS2#18-CERT#10) showed that three *CERT* gene alleles had frameshift-causing deletions (Figure 4A), and no CERT was detected by Western blot analysis (Figure 4B). Furthermore, SM synthesis was markedly reduced (Figure 5A and Figure S7A,B), indicating the complete loss of CERT in this mutant cell clone.

Next, the proportion of VLC-sphingolipids in the TAL-CERS2#18-CERT#10 clone was compared with that in the TAL-CERS2#18 clone. In the TAL-CERS2#18-CERT#10 clone, the proportion of VLC-Cer was significantly reduced (i.e., the proportion of C16:0-Cer was increased) compared with that in the parental TAL-CERS2#18 clone. This was consistent with the expectation that C16:0-Cer would be originally transported for SM synthesis and remain unused due to the deficiency of CERT (Figure 5B,C). Nevertheless, the proportion of VLC-GlcCer in the TAL-CERS2#18-CERT#10 clone was almost the same as that in its parental TAL-CERS2#18 clone (Figure 5A,C). These results excluded the aforementioned possibility. Thus, the quantitative balance between VLC-Cer and C16:0-Cer in the ER was unlikely to be the major reason for the VLC-preference of GlcCer.

The minor SM synthesis shown in the TAL-CERS2#18-CERT#10 clone is thought to involve a putative CERT-independent ceramide transport pathway (Figure 5A) [9,32]. The proportion of VLC-SM in this clone was similar to that of VLC-Cer and was higher than that from the CERT-dependent SM synthesis shown in the TAL-CERS2#18 clone (Figure 5A,C). However, the proportion of VLC species in GlcCer was still higher than that in SM even though both ceramide transport pathways are CERT-independent, suggesting that a yet-to-be-identified mechanism exists to recruit VLC-Cer to UGCG.

### 2.4. Reduction in the Proportion of VLC-GlcCer by Treatment with Brefeldin A and Overexpression of UGCG

There were still two other possibilities that could be a major cause for the VLC-Cer-preference of GlcCer. One is the substrate specificity of UGCG, and the other is the difference in accessibility of ceramide subspecies to UGCG. UGCG is mainly localized at the cis Golgi, whereas ceramides are synthesized at the ER. Therefore, when the Golgi apparatus was merged with the ER by treatment with Brefeldin A (BFA) [38,39], UGCG must have accessed ceramides more freely. If the proportion of VLC-GlcCer were maintained in the presence of BFA, the reason for the VLC-preference would be UGCG substrate specificity, whereas if the proportion of VLC-GlcCer were reduced, the reason for the VLC-preference would be the difference in accessibility of ceramide subspecies to UGCG. Therefore, we investigated the effect of BFA treatment on the VLC-preference of GlcCer. BFA treatment greatly increased the synthesis of both GlcCer and SM, but not ceramide, in all tested cell types probably because both UGCG and SMS1 could easily access the newly synthesized ceramides without the need of intermembrane ceramide transport (Figure S7). More importantly, the proportion of VLC-GlcCer was reduced to 30% in the TAL-CERS2#18 clone in this condition (Figure 5C). Thus, UGCG was capable of utilizing both VLC-Cer and C16:0-Cer when the enzyme could access these ceramide substrates. In other words, the VLC-Cer-preference of GlcCer was due to the accessibility of ceramide subspecies to UGCG.

**Figure 6:**
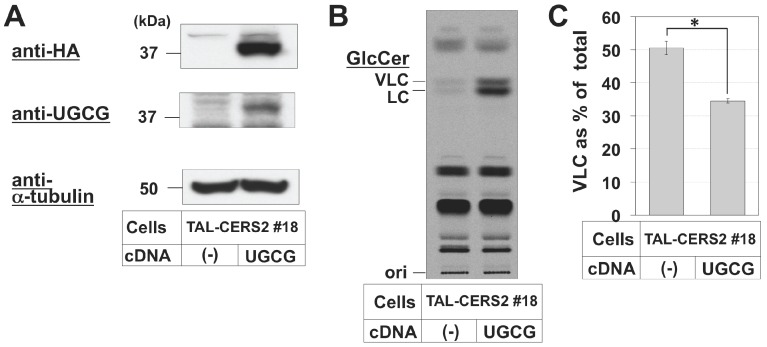
Effect of UGCG overexpression on the proportion of VLC in GlcCer. (**A**) Western blot analysis of UGCG in UGCG-overexpressed TAL-CERS2#18 cells. Cell lysates from TAL-CERS2#18 cells and UGCG-overexpressed TAL-CERS2#18 cells were immunoblotted with anti-HA antibody (HA), anti-UGCG antibody (**top**), and anti-α-tubulin antibody (**bottom**). 18, TAL-CERS2#18 clone; UGCG, UGCG overexpression. The whole images are shown in Figure S8. (-), no transfection; UGCG, UGCG cDNA overexpression; (**B**) metabolic labeling of sphingolipids with radioactive serine in TAL-CERS2#18 and UGCG-overexpressed TAL-CERS2#18 cells. Cells were cultured with [^14^C]serine for 2 h, and lipids extracted from the cells were separated by Method 1 for GlcCer; (**C**) comparison of the proportions of VLC-GlcCer between endogenous GlcCer in TAL-CERS2#18 cells and overexpressed GlcCer in UGCG-overexpressed TAL-CERS2#18 cells. The proportions of VLC-containing GlcCer are expressed as described in Figure 2B: mean percentage ± SD obtained from three independent experiments. The Student’s *t*-test was used. * *p* < 0.05.

In addition, we observed that overexpression of UGCG in the TAL-CERS2#18 clone also caused a reduction in the proportion of VLC-GlcCer (Figure 6). This was probably because some UGCG had leaked out of the restricted cis Golgi localization to other compartments, and the leaked UGCG could easily have accessed the newly synthesized ceramide, including C16:0-Cer, at the ER or other organelles, which is consistent with the result in the presence of BFA.

## 3. Discussion

In this study, we demonstrated that the proportion of VLC species to the total species in GlcCer was higher than that in SM using newly established CERS2-deficient HeLa cell mutants. These mutants included a null mutant type (TAL-CERS2#16) and cell lines that expressed different amounts of CERS2 (TAL-CERS2#18 and #13). A merit of TALEN is the ability to choose target sites more widely than the prevailing CRISPR/Cas system, and we chose the start codon of *CERS2* as the target site. The start codon was adjacent to the SA site of intron 1 (agGATG; the small letters indicate the SA site); therefore, the TALEN pair of CERS2 in this study also caused mutations of the SA site in some alleles of each mutant. As a result, exon skipping and SA shifting were observed in some CERS2 transcripts. Targeting the splicing donor or acceptor might have been a reasonable choice, especially if one attempted to disrupt multiallelic genes without regard to in-frame mutations in cancer cell lines. However, the splice site mutations often produced new splice variants; therefore, unusual types of mutants might have also been isolated. Indeed, the TAL-CERS2#18 clone lost the SA sites in all alleles, which was apparently a knockout cell line, but this clone still expressed a small amount of CERS2 from a transcript containing the wild-type open reading frame in the absence of a Kozak sequence at the 5′ side of the start codon. As a result, the expression level of the CERS2 in the TAL-CERS2#18 clone was much lower than that of TAL-CERS2#13, which had only one wild-type allele in four alleles.

The established mutant cell lines showed that the expression level of CERS2 was closely associated with the proportion of VLC acylation in sphingolipids, which was consistent with the previous reports [34,35,36]. Rather, in TAL-CERS2#18 and #13 clones, small amounts of CERS2 even seemed to raise the proportion of VLC-sphingolipids more than the apparent expression level. One possibility is that HeLa cells expressed abundant CERS2 and a partial reduction of the enzyme did not lead to the expected reduction of their metabolites. A similar phenomenon was also observed when CERS2 was reduced by an RNA interference experiment [40]. Another reason is that other CERSs affected the CERS2 activity. CERS5 is known to dimerize with CERS2 and promote the activity of CERS2, which may explain why the small amounts of CERS2 efficiently produced the VLC-sphingolipids [41,42].

The present study with several cognate CERS2-deficient HeLa cell mutants addressed the question of how the differential preference of ceramide acyl chain lengths between GlcCer and SM occurs. A schematic summary is shown in Figure S9. (1) VLC-Cer was preferentially used in GlcCer synthesis. Even when C16:0-Cer was enriched by disruption of the *CERT* gene, the high proportion of VLC-GlcCer was unchanged, suggesting that an unknown mechanism preferentially brings VLC-Cer to UGCG; (2) in contrast, C16:0-Cer was preferentially used in CERT-dependent SM synthesis, which was consistent with the previous in vitro studies showing that CERT transported C16:0-Cer more efficiently than VLC-Cer [43]. It should be emphasized that this does not mean that CERT is incapable of transporting VLC-Cer, because knockout of the *CERT* gene reduced the amount of VLC-Cer as well as C16:0-Cer [32]; (3) the proportion of VLC species in GlcCer was still higher than that of VLC species in SM synthesized in a CERT-independent manner. To date, it has not been intensively examined whether or not, in the absence of CERT, ceramide is transported to UGCG and SM synthases by the same mechanism. The data strongly suggest that at least two CERT-independent ceramide transport pathways exist: one is for SM and the other is for GlcCer; (4) the proportion of VLC-GlcCer decreased when UGCG functioned outside of the restricted region in BFA-treated cells as well as in UGCG-overexpressing cells, probably because UGCG accessed various ceramide species under these UGCG-mislocalizing conditions (Figure 5 and Figure 6).

A previous study showed that the synthesis of glycosylphosphatidylinositol (GPI)-anchored proteins affected the level of VLC-GlcCer but not VLC-SM [30]. The molecular mechanisms remain elusive of how ceramides are transported to UGCG or how UGCG encounters ceramides in mammals. Histochemical studies using epitope-tagged proteins suggest that UGCG is mainly localized at the cis face of the Golgi [8], whereas ceramide synthases including CERS2 are localized at the ER [14]. However, the histochemistry of their endogenous counterparts has not been studied. Previous studies showed that the transport of ceramides to UGCG is energy-independent, which suggests that the transport is vesicular independent [37,44], whereas other studies suggested that the CERT-independent transport of ceramide from the ER to the Golgi is vesicular dependent [11]. Further characterization of the high accessibility of VLC-Cer for UGCG would help to understand the CERT-independent ceramide transport pathways.

Previously, we constructed a “sphingolipid-related HeLa cell panel” including several sphingolipid-deficient HeLa mutant clones [32], and CERS2 mutants were added to the panel in this study. The length of FAs in sphingolipids affects cellular signaling including Lyn, a Src kinase [23,34] and insulin receptor [24]. The CERS2 mutants established in this study, especially TAL-CERS2#16, which lost its VLC-sphingolipids, may be useful to investigate mechanisms of how the FA acyl chain length in sphingolipids affects cellular phenomena and signaling as well as a mechanism of CERT-independent ceramide transport as described above.

## 4. Materials and Methods

### 4.1. Cell Culture, Antibodies, and Reagents

HeLa-mCAT#8 cells, which express mouse cationic amino acid transporter 1 (serves as the mouse ecotropic retroviral receptor) [45], and its CERS2 mutants were maintained in Dulbecco’s modified Eagle’s medium (DMEM) containing 10% fetal bovine serum (FBS). Mouse anti-α tubulin IgG (DM1A), horseradish peroxidase (HRP)-conjugated rat anti-hemagglutinin (HA) IgG (3F10), puromycin, brefeldin A, and lysenin were purchased from Sigma. Rabbit anti-CERS2 antibody was from Bethyl Laboratories (Montgomery, TX, USA). Rabbit anti-COL4A3BP (CERT) antibody (ab72536) was from Abcam (Cambridge, UK). Mouse anti-UGCG IgG (1E5) was from Abnova (Taipei, Taiwan). Blasticidin was from Wako (Tokyo, Japan). PrimeStar GXL (for cloning and sequencing) and EmeraldAmp PCR Master Mix (for PCR after reverse transcription) were from Takara Bio (Otsu, Japan). Zero Blunt TOPO PCR Cloning Kit was from ThermoFisher Scientific. TLC plates (Silica Gel 60), High-performance TLC (HPTLC) plates (Silica Gel 60), HPTLC aluminum sheets, and HPTLC RP-18 (C18 reverse phase TLC) plates were from Merck. l-[U-^14^C]serine (5.957 GBq/mmol) was from Moravek (Brea, CA, USA), and d-[1-^14^C]galactose (2.072 GBq/mmol) was from GE Healthcare. C16:0-, C20:0-, C24:0- and C24:1-ceramides were from Cayman Chemical (Ann Arbor, MI, USA). C18:0- and C22:0-ceramides were from Toronto Research Chemicals (Toronto, ON, Canada). All primers used for PCR are described in Supplemental Experimental Section 2.

### 4.2. Synthesis of TALEN Constructs

The original “Golden Gate TALEN and TAL Effector Kit”, developed by the Voytas group, was obtained through Addgene (Cambridge, MA, USA) [46]. Modified pTAL plasmids, pTAL-ModB, were constructed previously [32]. A CERS2 TALEN pair was designed using TALE-NT 2.0 web-based tools [47], and the repeat variable di-residues (RVDs) that were used are described in Supplemental Experimental Section 1. TALEN module assembly was performed as described previously and the assembled cassette was ligated into the multicloning site of pSELECT-puro-L1 [32]. The CERT TALEN pair (version 2) was constructed previously [32].

### 4.3. Subcloning of CERS2 and UGCG cDNAs in Retroviral Vectors and Their Retroviral Infection

The pMXs-IB–hCERS2 vector was constructed as follows: human *CERS2* cDNA was amplified by PCR (template: HeLa cDNA, primers: CERS2-EcoRI-ATGs and CERS2-XhoI-STOPas). The amplified DNA (DDBJ/EMBL/GenBank Accession number: NM_022075.4) was digested with EcoRI and XhoI, and inserted into pMXs-IB [48]. pMXs-IB–hUGCG-HA was constructed using Seamless Cloning and Assembly Kit (Thermo Fisher, Waltham, MA, USA) as follows: both human *UGCG* cDNA and the linearized pMXs-IB-cHA plasmid were amplified by PCR (hUGCG: template: HeLa cDNA, primers: UGCGrecATGs and UGCGrecEND-HAas; linearized pMXs-IB-cHA: template: pMXs-IB-GRINA-HA plasmid, which was constructed from pMXs-IP-GRINA-HA [45], primers: pMXs-IB-5’as and pMXs-HA-3’s). The two fragments were assembled by recombination using the kit described above. The preparation of retrovirus particles and their infection of HeLa cells were performed as described previously [45,48].

### 4.4. Genome editing with TALENs

Genome editing of HeLa cells using the TALEN technology was performed as described previously [32]. Briefly, HeLa–mCAT#8 cells or HeLa mutant cells were transfected with a pair of TALEN plasmids. After puromycin treatment to remove untransfected cells and incubation at 30 °C, the TALEN-treated HeLa cells were harvested for indel analysis or diluted to isolate gene-disrupted clones. In *CERT* gene knockout in TAL-CERS2#18 cells, the TALEN-CERT-treated cells were treated with lysenin, a cytolytic toxin targeting the SM-containing membrane, to concentrate the SM-reduced cells by *CERT* gene disruption. Indel analysis was performed with genomic PCR, TOPO cloning, and DNA sequences, as described previously [32].

### 4.5. mRNA Analysis

RNA isolation, reverse transcription, TOPO cloning and DNA sequences were performed as described previously [32]. For real-time PCR, the LightCycler system with the LightCycler-FastStart DNA master SYBR Green I kit (Roche, Penzberg, Germany) was used as described previously [45]. *GAPDH* was used as the housekeeping gene. The mean percentage ± SD was obtained from three independent experiments. In the RT-PCR analysis shown in Figure S4C, human testis cDNA (T) was synthesized using human testis mRNA (Thermo Fisher) and used as a positive control. Images of the RT-PCR ethidium bromide-stained agarose were acquired with Gel Doc EZ system (Bio-Rad, Hercules, CA, USA).

### 4.6. Metabolic Labeling of Sphingolipids and TLC Analysis

Metabolic labeling experiments using l-[U-^14^C]serine and d-[1-^14^C]galactose including mild alkaline methanolysis were performed as described previously [32,45]. Simply, cells were incubated with 18.5 kBq of l-[U-^14^C]serine or 6.1 kBq of d-[1-^14^C]galactose in Opti-MEM with 1% Neutridoma-SP (Roche) for 2 or 16 h. In the BFA treatment, the cells were pretreated with it for 45 min, and then labeling was performed in its presence. The cells were lysed with 0.1% sodium lauryl sulfate (SDS), and lipids in the lysate were extracted by the method of Bligh and Dyer [49]. Separation of the lipids by TLC was performed using four methods, as follows: (Method 1) plate: TLC60 plate (20 cm × 20 cm), developing solvent: methyl acetate/n-propanol/chloroform/methanol/0.25% KCl = 50/50/50/20/18 [50]. This method was used to observe [^14^C]serine-labeled GlcCer and SM biosynthesis. (Method 2) plate: HPTLC60 plate (10 cm × 20 cm), developing solvent: chloroform/methanol/0.25% CaCl_2_ = 65/35/8 [45]. This method was used to observe [^14^C]galactose-labeled GlcCer and Gb3 biosynthesis. (Method 3) plate: HPTLC60 plate (10 cm × 20 cm), developing solvent: toluene/chloroform/acetone = 7/3/3, development and desiccation three times. This method was used to observe [^14^C]serine-labeled ceramide biosynthesis. (Method 4) (1) Separation of ceramide from other lipids, plate: HPTLC60 aluminum sheet (20 cm × 20 cm), first developing solvent: methyl acetate/n-propanol/chloroform/methanol/0.25% KCl = 50/50/50/20/18, development to 14 cm from the origin. Second developing solvent: toluene/chloroform/acetone = 7/1/2, development to the top of the plate. Non-radiolabeled ceramide standards were simultaneously developed next to the samples; (2) extraction of the labeled ceramides. The standard ceramides were visualized by iodine vapor to visualize the position of ceramides, and then the TLC alumina pieces containing labeled ceramide were cut out. The radioactive ceramides were extracted from the TLC pieces by the method of Bligh and Dyer; (3) Separation of ceramide species by reverse-phase TLC. plate: HPTLC60 RP-18 (10 cm × 20 cm), developing solvent: chloroform/methanol/H_2_O = 8/15/1 [34]. Non-radiolabeled ceramides were simultaneously developed next to the samples, and visualized by iodine vapor. The positions of the spot origin and the solvent front in two images were aligned. This method was used to observe different FA lengths of [^14^C]serine-labeled ceramide. The radioactive lipids on TLC plates were visualized, and the intensity of each band was quantified using a Typhoon FLA 7000 (GE Healthcare). To visualize the results more clearly, some images were processed with Gimp software to obtain high contrast images. Note that quantification was performed using only the original images visualized by Typhoon FLA 7000. To compare the relative amounts of the lipids, the total band intensity (the sum of upper and lower band intensities) of each sphingolipid in the parent cells (HeLa-mCAT#8) was taken as 100%. To compare the proportion of the upper band intensity in the indicated lipids (the proportion of VLC-containing lipids), the total band intensity (the sum of upper and lower band intensities) of each lipid in the indicated cells was taken as 100%. The mean percentage ± SD was obtained from at least three independent experiments.

### 4.7. Western Blot

Lysate preparation (radioimmunoprecipitation assay (RIPA) buffer) and Western blot analysis were performed as described previously [45]. Briefly, HeLa–mCAT#8 cells or HeLa mutant cells were lysed with RIPA buffer. The postnuclear fraction was resolved by SDS-PAGE, transferred to polyvinylidene difluoride (PVDF) membranes using a wet transfer method, and probed with the antibodies described in Section 4.1. Rabbit anti-CERS2 recognizes a region between residues 330 and 380 of human CERS2 (NP_071358.1). Rabbit anti-CERT recognizes a region between residues 300 and 350 of human CERT (NP_005704.1).

### 4.8. Cell Growth

Cells (2.5 × 10^4^ in 12-well plates or 5 × 10^4^ in 6-well plates) were cultured at 37 °C for one and four days, respectively, and the cell number was counted by a hemocytometer. The growth rate was expressed as the fold change calculated by (cell number on Day 4)/(cell number on Day 1). The mean fold ± SD was obtained from six independent experiments.

### 4.9. Statistical Analysis

The Student’s *t*-test was used for statistical analysis, setting *p* < 0.05 as a statistical significance criterion. For multiple comparison, the Student’s *t*-test with Bonferroni correction was used, setting *p* < 0.017 (0.05 divided by 3) as a statistical significance criterion when three samples were compared with each other and *p* < 0.025 (0.05 divided by 2) as a statistical significance criterion when one sample was compared with two samples.

## 5. Conclusions

We here established several *CERS2*-edited HeLa cell clones with different proportions of VLC-Cer. Metabolic labeling of de novo synthesized sphingolipids with radioactive serine demonstrated that the VLC-type proportion in GlcCer was higher than that in SM for any expression level of CERS2. In addition, the higher proportion was still maintained even when the CERT-dependent pathway to convey C16-rich ceramides to the Golgi site of SM synthesis was destroyed. On the other hand, when the Golgi apparatus was merged with the ER by BFA, the synthetic rate of GlcCer expression was greatly enhanced with a reduced proportion of its VLC-type, which suggested that UGCG was capable of recognizing C16-Cer as well, but that CERT-independent delivery of ceramides from the ER to the site of GlcCer synthesis was rate-limiting in the de novo synthesis of GlcCer. Based on these results, we would like to propose that intracellular ceramide logistics, which renders VLC-Cer more accessible than C16-Cer to the UGCG, exists in mammalian cells.

## Supplementary Materials

Supplementary materials can be found at www.mdpi.com/1422-0067/17/10/1761/s1.
