# Supplementary material for: Role of Intracellular Lipid Logistics in the Preferential Usage of Very Long Chain-Ceramides in Glucosylceramide
Source: Int J Mol Sci. 2016 Oct 21;17(10):1761. doi: 10.3390/ijms17101761 (PMC5085785; doi:10.3390/ijms17101761)
Supplement: Supplementary file 1 [file ijms-17-01761-s001.pdf]

Supplementary Materials: Role of Intracellular Lipid Logistics in the Preferential Usage of Very Long Chain-Ceramides in Glucosylceramide

Toshiyuki Yamaji, Aya Horie, Yuriko Tachida, Chisato Sakuma, Yusuke Suzuki, Yasunori Kushi and Kentaro Hanada

1. Repeat Variable di-Residue Sequences in the TALENs Used in this Study

TALEN-CERS2	Fw: NN-HD-HD-HD-NG-HD-HD-HD-HD-NG-NG-NG-NN-HD-HD-HD-HD-NG-HD<br>Rv: NI-NI-NG-HD-NI-NG-NI-HD-NI-NI-NN-NN-NG-HD

2. Used Primers

Indel Analysis in *CERS2* Gene

CERS2 s1: GATCTAGAGTTCGACTCTCTGCC

CERS2 s2: GTGATTCCGGCTCACTGCAACC

CERS2 as1: CAGCGTGATATAGAGATCTGAGGCT

CERS2 as2: AGCTGCCTGTGTGTTCACTAGTGG

Cloning of *CERS2* and *UGCG* cDNAs

CERS2-EcoRI-ATG s:

ACC<u>GAATTC</u>AGG**ATG**CTCCAGACCTTGTATGATTACTTC (underlined: EcoRI site; bold: start codon)

CERS2-XhoI-STOP as:

ACC<u>CTCGAG</u>**TCA**GTCATTCTTACGATGGTTGTTATTGAGGATG (underlined: XhoI site; bold: stop codon)

UGCGrecATG s:

<u>GTTAATTAAGGATCC</u>AAG**ATG**GCGCTGCTGGACCTGG (underlined: sequence for recombination; bold: start codon)

UGCGrecEND-HA as:

<u>GACGTCATATGGGTA</u>TACATCTAGGATTTCCTCTGCTGTACC (underlined: sequence for recombination; bold: part of HA-tag sequence)

pMXs-IB-5′ as:

<u>GGATCCTTAATTAAC</u>TAGCTAGATCCGG (underlined: sequence for recombination)

pMXs-HA-3′ s:

<u>TACCCATATGACGTC</u>CCGGACTA (underlined: sequence for recombination; bold: part of HA-tag sequence)

RT-PCR of *CERS1-6* mRNAs

CERS1 s: TACGCTATACATGGACACCTGGC

CERS1 as: GGGACTTGAAGTAAATGTTGAGCTTGG

CERS2 s: AGTCTCCTCAAGAAGTTCCGAGAAG

CERS2 as: TGTTCCTTGAAATCCTTTCGCTTGACATC

CERS3 s: AGCTCATATCATCCACCACCTGG

CERS3 as: GGAAAAACAATGAGGCGGCTGATG

CERS4 s: TGTGAGGGATCAGACCAGGAG

CERS4 as: CAGCTGGCCTCACAGAACTTC

CERS5 s: CTGTCAAAGCAGCTGGATTGGAATG

CERS5 as: GGATAGTTATGCCAGCACTGTCG

CERS6 s: CCATTCTGGAAAAGGTCTTCACTGC

CERS6 as: GTAGTGAAGGTCAGTTGTGAGTGG

CERS2 5′ UTR s: GAGCAGACGGAGTACACGGA

CERS2 Ex4 as: ACGGAACCAACGCTCTACCTG

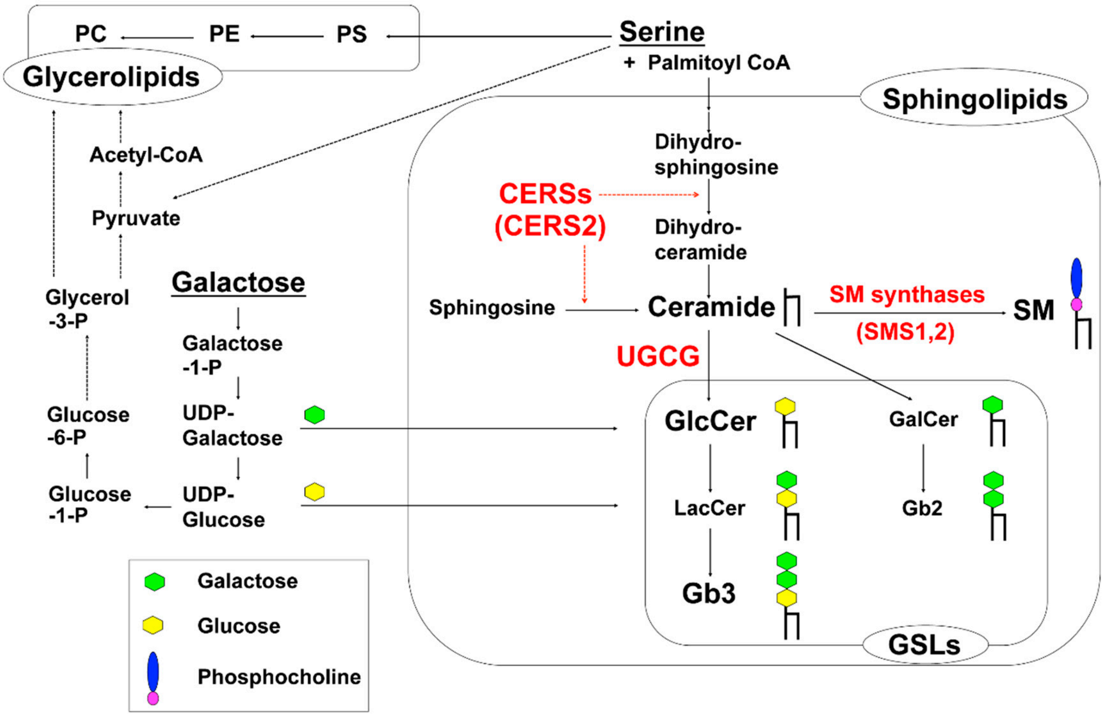

Figure S1. Metabolism of sphingolipids. The biosynthetic pathway of sphingolipids relevant to this study is shown. Serine is incorporated into both sphingolipids and glycerolipids. Galactose is mainly incorporated into glycosphingolipids (GSLs), but galactose is also converted to glycerol-3-phosphate (glycerol-3-P) and acetyl-CoA to be incorporated into glycerolipids. Red letters indicate the enzymes involved in this study. SM, sphingomyelin; GlcCer, glucosylceramide; LacCer, lactosylceramide; Gb3, globotriaosylceramide; GalCer, galactosylceramide; Gb2, galabiosylceramide (Galα1-4GalCer); PS, phosphatidylserine; PE, phosphatidylethanolamine; PC, phosphatidylcholine; CERS, ceramide synthase; SMS, sphingomyelin synthase; UGCG; UDP-glucose: ceramide glucosyltransferase (GlcCer synthase); GSLs; glycosphingolipids; P, phosphate.

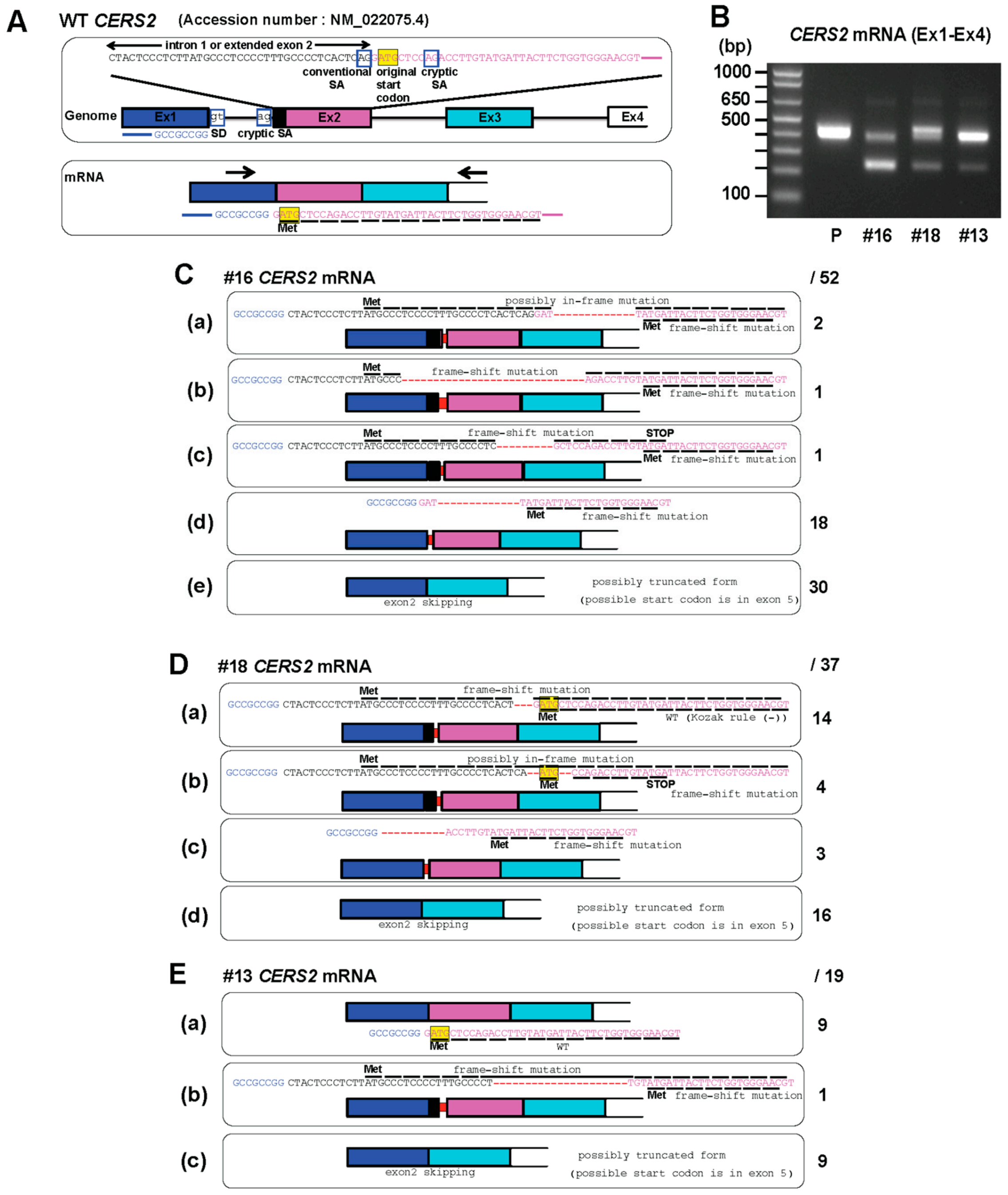

Figure S2. CERS2 transcripts expressed in TAL-CERS2 clones. (**A**) Schematic diagram of the genome and transcript of the wild-type *CERS2* gene. Arrows indicate the primers used in RT-PCR analysis in (**B**). Blue box, exon 1; pink box, exon 2; light blue box, exon 3; white box, exon 4; black box, extended exon 2; blue line box, splicing donor and acceptor; yellow box, start codon; (**B**) RT-PCR analysis of CERS2 in CERS2-deficient mutants. Primers bound to the 5′ untranslated region (5′ UTR) (exon 1) and exon 4 of CERS2 were used. P, parent cell line; 16, TAL-CERS2#16 clone; 18, TAL-CERS2#18 clone; 13, TAL-CERS2#13 clone. Note that a short transcript was observed in the mutants but not the parent cells, which indicates the exon 2-skipped transcript shown in (**C**–**E**); (**C**–**E**) schematic diagrams of CERS2 transcripts observed in (**C**) TAL-CERS2#16 clone, (**D**) TAL-CERS2#18 clone, and (**E**) TAL-CERS2#13 clone. The numbers of CERS2 transcripts analyzed in TAL-CERS2 clones are specified on the right of the sequences. Transcript D (a) contains the wild-type CERS2 open reading frame, but it does not follow the Kozak rule because the critical -3 position from the start codon was guanine in the wild-type transcript, whereas it was changed to cytosine in the transcript C (a) (CTGATG)). Colored boxes indicate the same as (**A**). Red dashes and boxes indicate deletions.

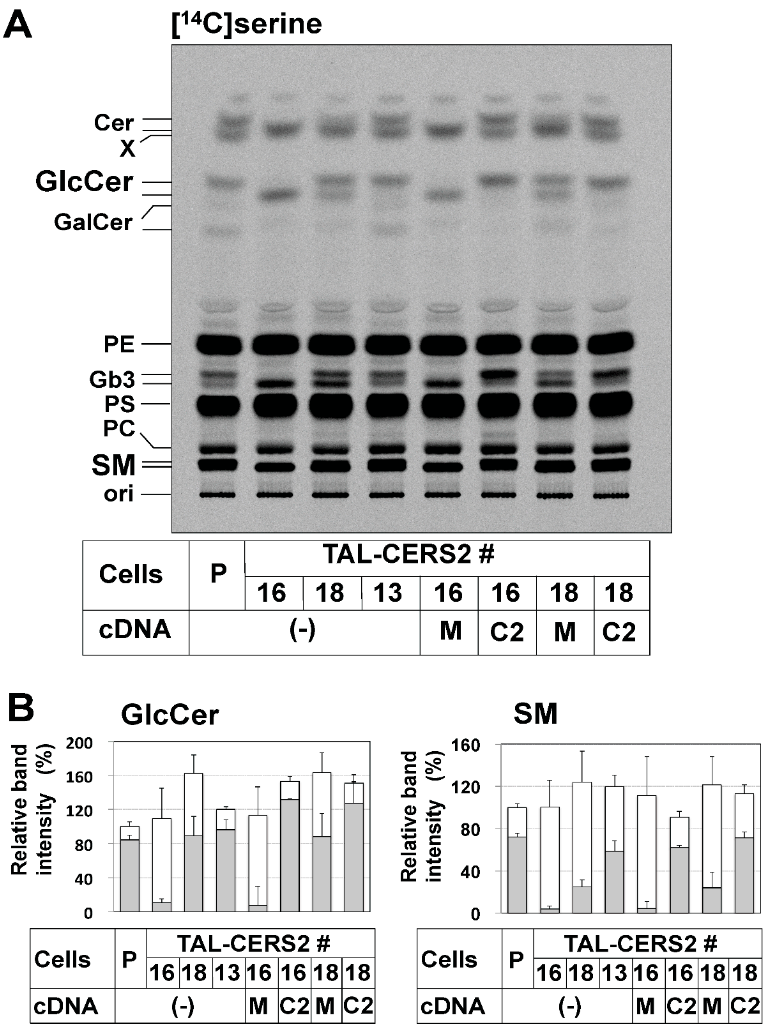

Figure S3. Metabolic labeling of lipids in TAL-CERS2 clones. (**A**) Metabolic labeling of sphingolipids with radioactive serine in the parent cells and TAL-CERS2 clones. The image is the darker one of Figure 2A. P, parent cell line; 16, TAL-CERS2#16 clone; 18, TAL-CERS2#18 clone; 13, TAL-CERS2#13 clone; (-), no transfection; M, mock; and C2, CERS2 cDNA restoration; (**B**) quantification of the [^{14}C]serine-labeled GlcCer and SM shown in Figure 2A. The relative amounts of the labeled lipids are expressed for each band intensity as the percentage of the sum of the upper and lower band intensities of GlcCer and SM in the parent cells. White columns indicate the percentage of lower band intensities (C16 LC-containing lipids), whereas gray columns indicate the percentage of upper band intensities (VLC-containing lipids): mean percentage ± SD obtained from five independent experiments.

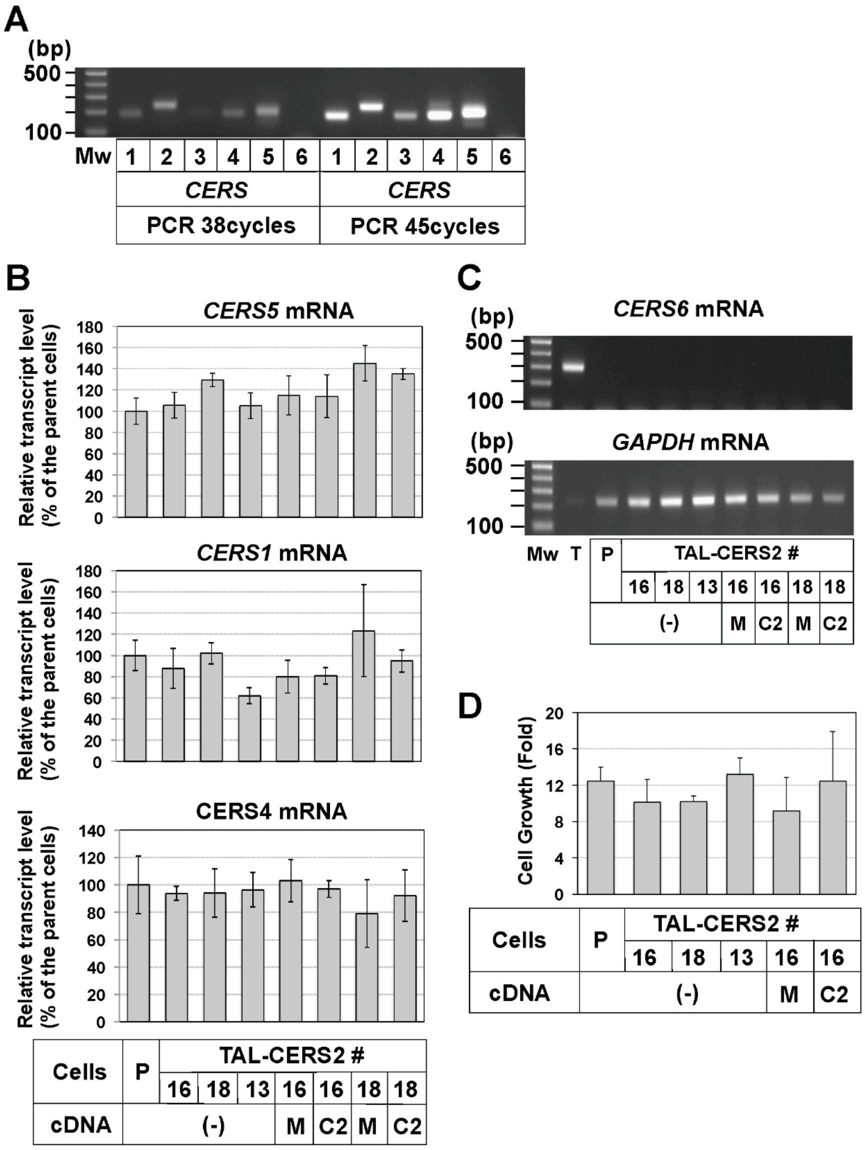

Figure S4. CERSs transcript analyses. (**A**) RT-PCR of CERS1–6 mRNAs in the parent HeLa cells; (**B**) quantitative real-time PCR of CERS1, 4, and 5 mRNA in the parent cells and TAL-CERS2 clones. The relative expression levels of CERS1, 4, and 5 mRNA to GAPDH mRNA in the parent cells were expressed as 100%. P, parent cell line; 16, TAL-CERS2#16 clone; 18, TAL-CERS2#18 clone; 13, TAL-CERS2#13 clone; (-), no transfection; M, mock; C2, CERS2 cDNA restoration; (**C**) RT-PCR analysis of CERS6 in the parent cells and TAL-CERS2 clones. Human testis cDNA was used as a positive control. No CERS6 was detected in 45 PCR cycles in all established mutants; (**D**) cell growth analysis. Cell numbers at Days 1 and 4 were counted and the fold change was calculated by (cell number on Day 4)/(cell number on Day 1). The mean fold ± SD was obtained from six independent experiments.

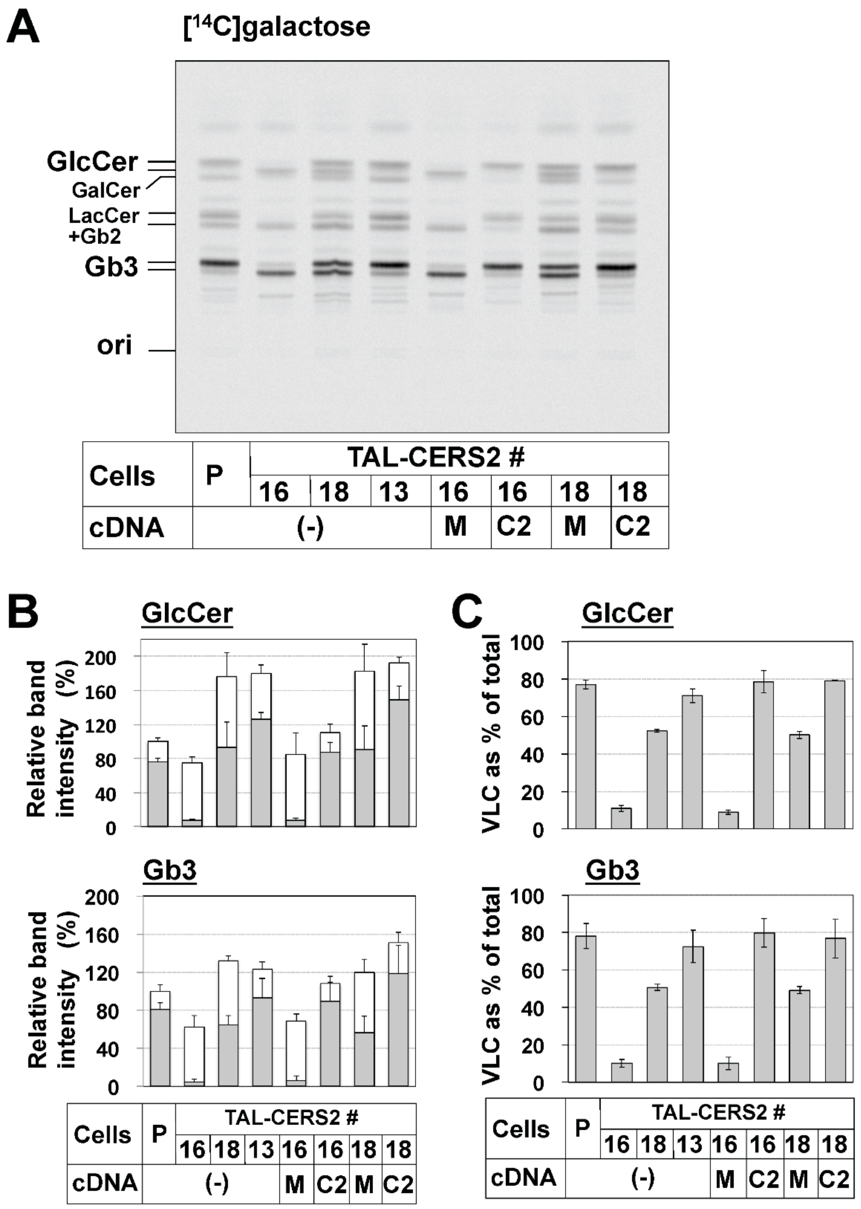

Figure S5. Biosynthetic analysis of GSLs in TAL-CERS2 clones. (**A**) Metabolic labeling of GSLs with radioactive galactose in the cells shown in Figure 1B. Cells were cultured with [14C]galactose for 16 h, and lipids extracted from the cells were treated with mild alkaline to remove glycerolipids and were separated by TLC (Method 2). A radioactive image of an analyzed TLC plate is shown. Cer, ceramide; GalCer, galactosylceramide; PE, phosphatidylethanolamine; PS, phosphatidylserine; PC, phosphatidylcholine; P, parent cell line; 16, TAL-CERS2#16 clone; 18, TAL-CERS2#18 clone; 13, TAL-CERS2#13 clone; (-), no transfection; M, mock; C2, CERS2 cDNA restoration; (**B**) quantification of the [^{14}C]galactose-labeled GlcCer and Gb3 shown in (A). The relative amounts of the labeled lipids are expressed for each band intensity as the percentage of the sum of the upper and lower band intensities of GlcCer and Gb3 in the parent cells. White columns indicate the percentage of lower band intensities (C16 LC-containing lipids), whereas gray columns indicate the percentage of upper band intensities (VLC-containing lipids); (**C**) the proportions of VLC-GlcCer and Gb3 shown in A and B. These are expressed as the percentage of the upper (VLC) band intensity of the sum of the upper (VLC) and lower (LC) band intensities of GlcCer and Gb3 for each cell line: mean percentage ± SD obtained from three independent experiments.

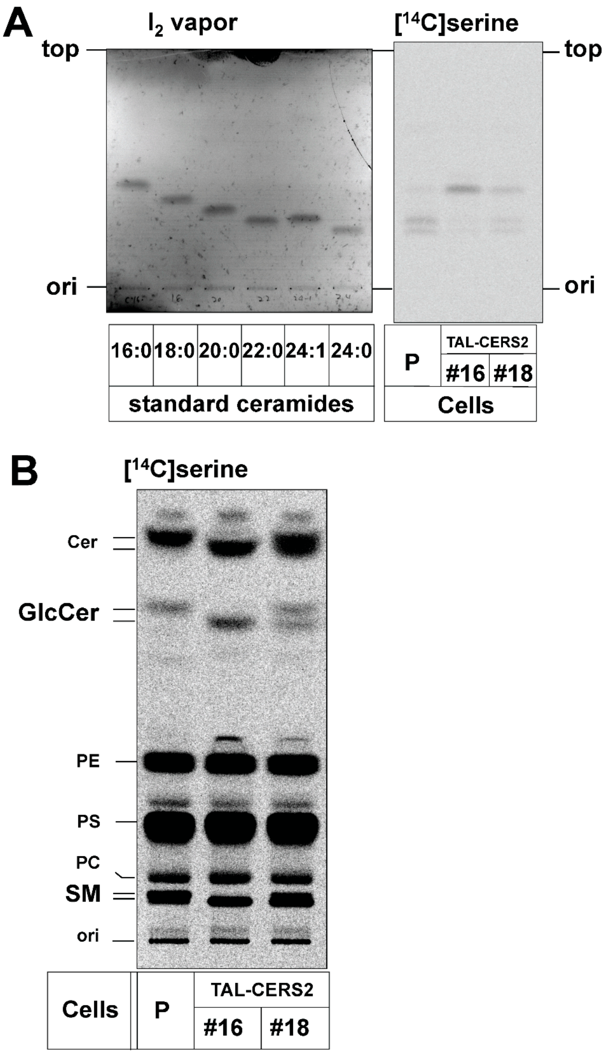

Figure S6. Different fatty acid preferences of GlcCer and SM. (**A**) Separation of ceramide subspecies containing different fatty acids by the combination of normal-phase and reverse-phase TLC according to Method 4. Non-radiolabeled ceramide standards (C16:0, C18:0, C20:0, C22:0, C24:1, and C24:0) were developed simultaneously and visualized by iodine vapor. The positions of the spot origin (ori) and the solvent front (top) in two images were aligned; (**B**) metabolic labeling of sphingolipids with radioactive serine in CERS2-deficient mutants. The image is the darker one of Figure 3B.

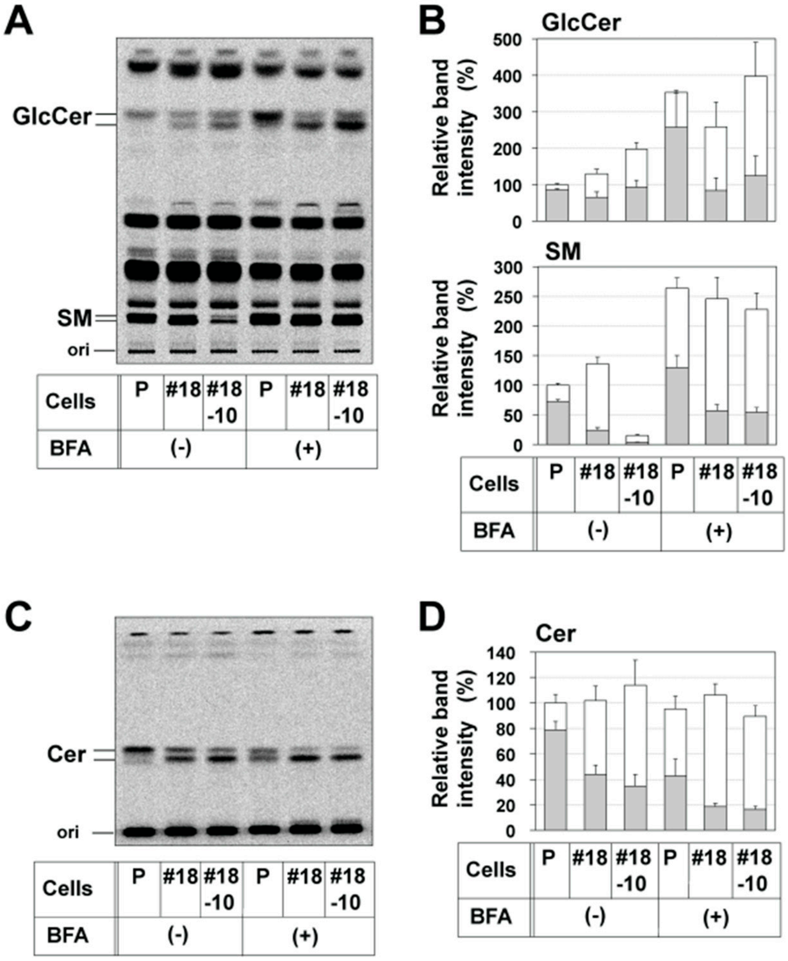

Figure S7. Effects of CERT disruption and BFA treatment on the proportion of VLC species in sphingolipids. (**A**,**C**) Metabolic labeling of sphingolipids with radioactive serine in the parent cells (P), TAL-CERS2#18 clone (#18), and TAL-CERS2#18-CERT#10 clone (#18-10). Cells were cultured with [^{14}C]serine in the presence (+) or absence (-) of BFA for 2 h. These images are the darker ones of Figure 5A,B; (**B**,**D**) quantification of the [^{14}C]serine-labeled GlcCer and SM shown in Figure 5A and the [^{14}C]serine-labeled ceramide shown in Figure 5B. The method was as described in Figure S3B.

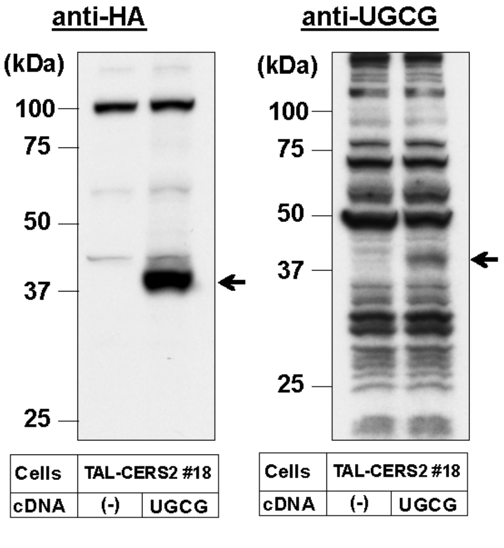

Figure S8. Western blot analysis of UGCG in UGCG-overexpressed TAL-CERS2#18 cells. These images are the whole ones of Figure 6A. (-), no transfection; UGCG, UGCG cDNA overexpression.

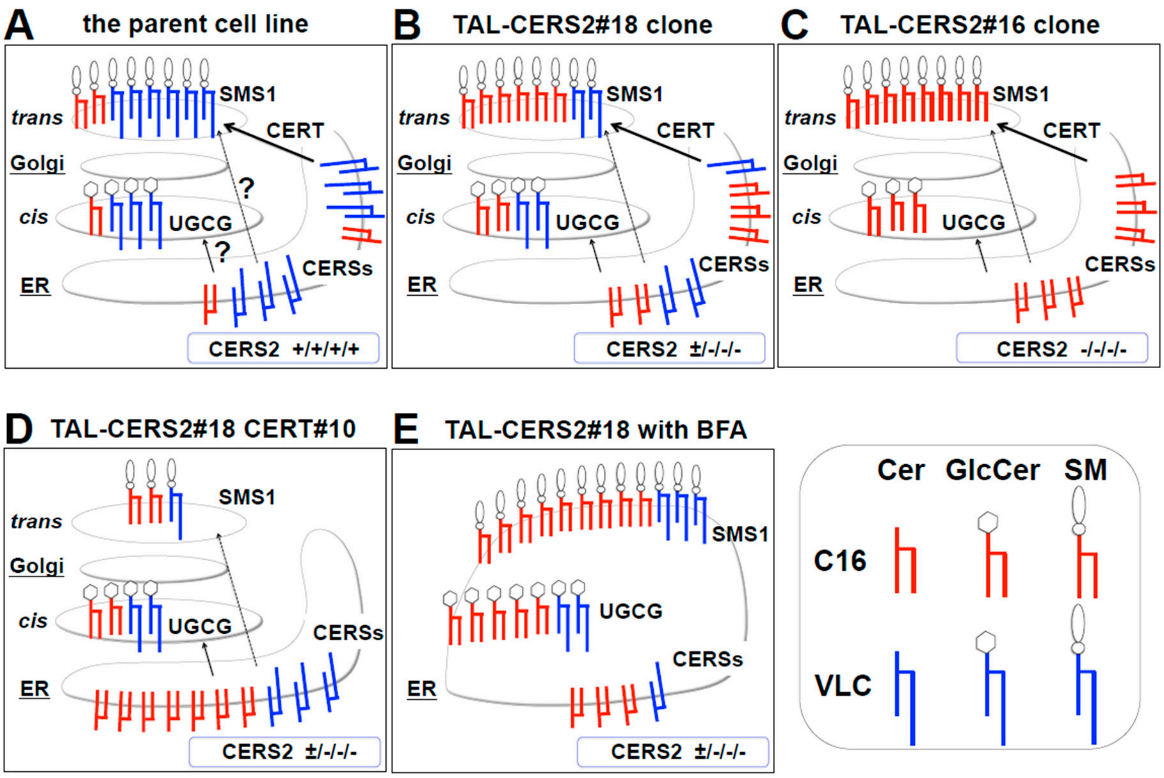

Figure S9. Schematic summary of the proportions of VLC- and C16-containing sphingolipids in TAL-CERS2 clones. (**A**) The parent cell lines; (**B**) TAL-CERS2#18 clone; (**C**) TAL-CERS2#16 clone; (**D**) TAL-CERS2#18-CERT#10 (#18-10) clone; (**E**) BFA-treated TAL-CERS2#18 clone. Red, C16-Cer; Blue, VLC-Cer; hexagon, glucose; oval with circle, phosphocholine. The symbol "?" indicates unidentified factor(s) that are involved in CERT-independent ceramide transport pathways.
